# Discrimination of Radiologists' Experience Level Using Eye-Tracking Technology and Machine Learning: Case Study

**DOI:** 10.2196/53928

**Published:** 2025-01-22

**Authors:** Stanford Martinez, Carolina Ramirez-Tamayo, Syed Hasib Akhter Faruqui, Kal Clark, Adel Alaeddini, Nicholas Czarnek, Aarushi Aggarwal, Sahra Emamzadeh, Jeffrey R Mock, Edward J Golob

**Affiliations:** 1 Department of Mechanical Engineering The University of Texas at San Antonio San Antonio, TX United States; 2 Department of Engineering Technology Sam Houston State University Huntsville, TX United States; 3 Department of Radiology University of Texas Health Science Center at San Antonio San Antonio, TX United States; 4 Department of Mechanical Engineering Southern Methodist University Dallas, TX United States; 5 Department of Psychology The University of Texas at San Antonio San Antonio, TX United States

**Keywords:** machine learning, eye-tracking, experience level determination, radiology education, search pattern feature extraction, search pattern, radiology, classification, gaze, fixation, education, experience, spatio-temporal, image, x-ray, eye movement

## Abstract

**Background:**

Perception-related errors comprise most diagnostic mistakes in radiology. To mitigate this problem, radiologists use personalized and high-dimensional visual search strategies, otherwise known as search patterns. Qualitative descriptions of these search patterns, which involve the physician verbalizing or annotating the order he or she analyzes the image, can be unreliable due to discrepancies in what is reported versus the actual visual patterns. This discrepancy can interfere with quality improvement interventions and negatively impact patient care.

**Objective:**

The objective of this study is to provide an alternative method for distinguishing between radiologists by means of captured eye-tracking data such that the raw gaze (or processed fixation data) can be used to discriminate users based on subconscious behavior in visual inspection.

**Methods:**

We present a novel discretized feature encoding based on spatiotemporal binning of fixation data for efficient geometric alignment and temporal ordering of eye movement when reading chest x-rays. The encoded features of the eye-fixation data are used by machine learning classifiers to discriminate between faculty and trainee radiologists. A clinical trial case study was conducted using metrics such as the area under the curve, accuracy, *F*_1_-score, sensitivity, and specificity to evaluate the discriminability between the 2 groups regarding their level of experience. The classification performance was then compared with state-of-the-art methodologies. In addition, a repeatability experiment using a separate dataset, experimental protocol, and eye tracker was performed with 8 participants to evaluate the robustness of the proposed approach.

**Results:**

The numerical results from both experiments demonstrate that classifiers using the proposed feature encoding methods outperform the current state-of-the-art in differentiating between radiologists in terms of experience level. An average performance gain of 6.9% is observed compared with traditional features while classifying experience levels of radiologists. This gain in accuracy is also substantial across different eye tracker–collected datasets, with improvements of 6.41% using the Tobii eye tracker and 7.29% using the EyeLink eye tracker. These results signify the potential impact of the proposed method for identifying radiologists’ level of expertise and those who would benefit from additional training.

**Conclusions:**

The effectiveness of the proposed spatiotemporal discretization approach, validated across diverse datasets and various classification metrics, underscores its potential for objective evaluation, informing targeted interventions and training strategies in radiology. This research advances reliable assessment tools, addressing challenges in perception-related errors to enhance patient care outcomes.

## Introduction

Lung cancer is the leading cause of cancer death, claiming 139,000 American lives yearly [1]. To mitigate its impact, the US Preventative Task Force recommends annual radiological screening for at-risk individuals [2]. Radiologists identify suspicious lung lesions (nodules) from patient chest images and recommend further management, including biopsy, continued surveillance, or further workup. Radiological surveillance reduces population mortality from lung cancer, but it is estimated that radiologists will make errors on 33% of abnormal chest exams, eliminating the chance for patients to start lifesaving treatment [3]. The predominant source of these errors is not deficient medical knowledge. Rather, errors primarily stem from the methods radiologists use to visually inspect the image, referred to as perceptual errors [4]. In other words, perceptual errors in radiology are mistakes that occur during the visual inspection and interpretation of medical images. They are distinct from cognitive errors, which involve incorrect reasoning or decision-making based on observed information. There are 2 primary patterns for overlooking a disease due to perceptual errors:

- Examining the affected area but ignoring the disease: This occurs when the radiologist inspects the region with the abnormality but fails to recognize it, possibly due to subtle presentation, distractions, or visual fatigue.
- Not examining the affected area: This happens when the radiologist misses the region with the abnormality entirely, often due to inefficient search patterns, incomplete scanning, or being misled by more prominent findings elsewhere.

Kundel [5] investigated the effects of perceptual errors in radiology and concluded that decisions and outcomes improve when radiologists’ experiences are enhanced.

Radiologists and radiology educators understand the stakes associated with missed diagnoses due to perceptual errors but have limited tools to combat these errors. Classical educational texts include general concepts, for example, “...scan the areas of least interest first, working toward the more important areas” [6], which, unfortunately, are inadequate to improve radiologist performance meaningfully.

Eye-tracking technology has been previously proposed as a tool to evaluate radiologist perception. Eye trackers are powerful because they provide high (>30 Hz) temporal and spatial resolution (approximately 1 degree of error). With the aid of eye tracking, quantitative analyses can be performed to understand the cognitive and perceptual processes better. Eye-tracking technology has previously proven relevant in evaluating decision-making processes [7], attention interruption [8], skill level determination [9], and impact of search pattern education [10].

In 2017, van der Gijp et al [11] performed a systematic literature review outlining the current state of science concerning visual perception in radiology. A key tenet is the global-focal search model [12-14], which can be summarized as the generation of an initial, fast global impression followed by a more detailed focal search. Eye-tracking technology allows these principles to be tested and potentially optimized to evaluate all clinically relevant portions of the exam in greater detail. Of the 22 relevant articles van der Gijp et al [11] reviewed, a consensus “traditional” feature set consisting of 5 features that could be experimentally measured was found to be associated with expertise.

Despite the development of this consensus feature set, visual search complexity may not be adequately captured by simple, low-dimensional features that do not fully describe how visual perception relates to skill. Machine learning is well-suited to provide deeper insight into radiologist visual search behavior and how this relates to radiologist performance. Waite et al [3] highlighted the importance of understanding perceptual expertise in radiology and the potential use of eye-tracking and perceptual learning methods in medical training to improve diagnostic accuracy. Lim et al [15] identified several features that can be extracted from eye-tracking data, including pupil size, saccade, fixations, velocity, blink, pupil position, electrooculogram, and gaze point, to be used in machine learning models. Among these features, fixation was the most commonly used feature in the studies reviewed.

Shamyuktha et al [16] developed a machine learning framework using eye gaze data such as saccade latency and amplitude to classify expert and nonexpert radiologists. Harezlak et al [17] investigated eye movement traits to differentiate experts and laymen in a similar study. Akshay et al [18] proposed a machine learning algorithm to identify eye movement metrics using raw eye-tracking data. Rizzo et al [19] used machine learning to detect cognitive interference based on eye-tracking data. Öder et al [20] applied machine learning to classify familiar web users based on eye-tracking data. Indeed, these techniques can be used to enhance competency assessment and feedback techniques in radiologists.

Eye tracking also holds the potential for understanding the longitudinal aspects of competency progression in medical education, allowing for examining how interpretive and diagnostic skills develop over time. Karargyris et al [21] and Bigolin Lanfredi et al [22] created and validated chest x-ray datasets with eye-tracking data and report dictation for developing such artificial intelligence systems. These datasets aim to support the research community in developing more complex support tools for radiology research.

In this study, we use machine learning to compare the discriminability of 2 radiologists of different skill levels using, first, the aforementioned “traditional” gaze-based features (such as time to scan, saccade length, the total number of fixations, and total regressive fixations) [11] and second, the “proposed” features that we developed to describe high-dimensional visual search patterns thoroughly and quantitatively. We curate the traditional feature sets to those that could be practically acquired without laborious manual ground truthing of exams, as this would permit large-scale deployment of this technology to health care institutions. To highlight the use of eye-tracking data and artificial intelligence, we term our general approach “biometric radiology artificial intelligence.”

**Figure 1:**
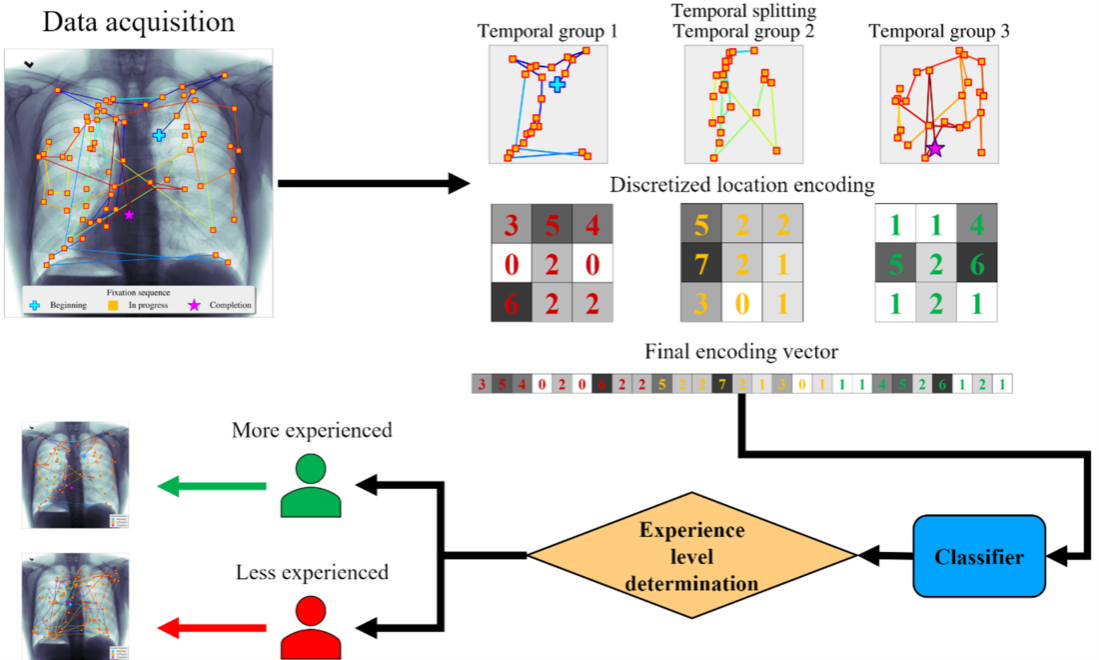
Overall algorithm: the steps required to generate proposed features from the raw dataset and build the proposed machine learning model.

The driving hypothesis behind the work presented in this paper is that gaze patterns measurably differ among radiologists as a function of their experience level. To test this hypothesis, we proposed a novel discretized feature encoding method that condenses fixation data into a few representative spatiotemporal bins for descriptive and predictive analytics purposes (Figure 1). With spatiotemporal binning, fixations are divided into a predefined number of temporal segments (bins). Within each temporal bin, the fixations are counted within spatial subdivisions of the image. This process results in a vector that captures detailed and structured information about both where and when fixations occurred. By splitting fixations into temporal bins, we capture the evolution of the visual search process over time, providing insights into how radiologists allocate their attention during different phases of image inspection. Also, spatial binning allows us to understand which regions of the image are being focused on and how frequently. In addition, this method transforms raw fixation data into structured features that can be effectively used by machine learning models.

We collected the gaze fixation data from radiologists while they were reading the x-rays. These data were then segmented into fixed temporal groups before discretizing them to convert them into final encoded vectors. The final encoded features were then used in training machine learning algorithms to classify radiologists.

Collecting data from 2 participants—1 faculty member (expert) and 1 resident (trainee)—we analyzed their behavior and level of experience using the proposed approach. Using stratified cross-validation over 10 folds, we compared the area under the curve (AUC) performance of several classifiers using the proposed methodology with the AUC performance of those same classifiers when using a traditional feature set (Table 1). We then confirmed our results using data from a second similarly designed, larger study evaluating 8 participants—4 faculty members (expert) and 4 residents (trainee). The remainder of the paper is structured as follows: *Methods* presents the data collection and preparation procedures and details of the proposed method; *Results* describes the simulation study and interpretation; and *Discussion* presents the discussion, concluding remarks, and advice to practitioners.

**Table 1:** State-of-the-art features from 22 relevant studies.

Attribute (per trial)	Attribute description	Association with high level of expertise (percentage of the total number of included studies)
Total time to scan	Measures the total duration spent scanning the chest x-ray image, indicating the thoroughness of the visual inspection	Decrease (45.45%)
Regressive fixation count	Counts the number of distinct locations revisited during the scan, suggesting areas of uncertainty or interest	Increase (4.55%) or decrease (4.55%)
Fixation count	Total number of fixations, reflecting the intensity of the visual scrutiny	Decrease (18.18%)
Total saccade length	Sum of all saccade lengths, indicating the extent and pattern of the visual search (time between fixations) [23] captured in a single chest x-ray scan	Increase (9.09%) or decrease (4.55%)
Coverage	Percentage of salient regions covered by the gaze, reflecting the comprehensiveness of the examination	Increase (9.09%) or decrease (9.09%)

## Methods

### Study Design, Data Collection, and Preparation

#### Overview

The study design was prospective, controlled, block-randomized, and Institutional Review Board (IRB) approved. Each study participant completed 4 roughly 1-hour sessions in a radiology reading room, including tutorial, calibration, assessment, and annotation periods. The tutorial included an overview of the assessment period and instructions on how to perform dictation and annotation consistently. Calibration was performed to ensure that recorded and actual gaze were consistent based on a 9-point custom calibration mapping script.

Nodule and normal cases were derived from the Shiraishi 2000 chest radiograph dataset [24], which includes 154 chest radiographs with 5 degrees of subtlety from level 1 (extremely subtle) to level 5 (obvious). Distractor cases were derived from the VinDr chest radiograph dataset [25]. A total of 3 sets of 6 nodule cases from the Japanese Society of Radiological Technology dataset, 1 set each from the intermediate difficulty levels (2, 3, and 4), and 1 set of 9 normal cases from the Japanese Society of Radiological Technology dataset were randomly sampled without replacement. In total, 2 cases each of pneumothorax, cardiomegaly, and consolidation from the VinDr dataset were randomly sampled without replacement to serve as distractor cases. These distractor cases functioned mainly to prevent control subject bias to the nodule detection task. Each participant reviewed the exams only once during the trial, and all study participants reviewed the same set of cases.

A custom software tool was developed to automatically display the study images and capture time-stamped bilateral gaze, bilateral pupil, head pose, voice, annotation, and image display configuration data. No chin rest was used to ensure that the study was performed in a manner that was as close as possible to a clinical setting. After each session, data were transferred to a database for further analysis.

#### Data Acquisition

**Figure 2:**
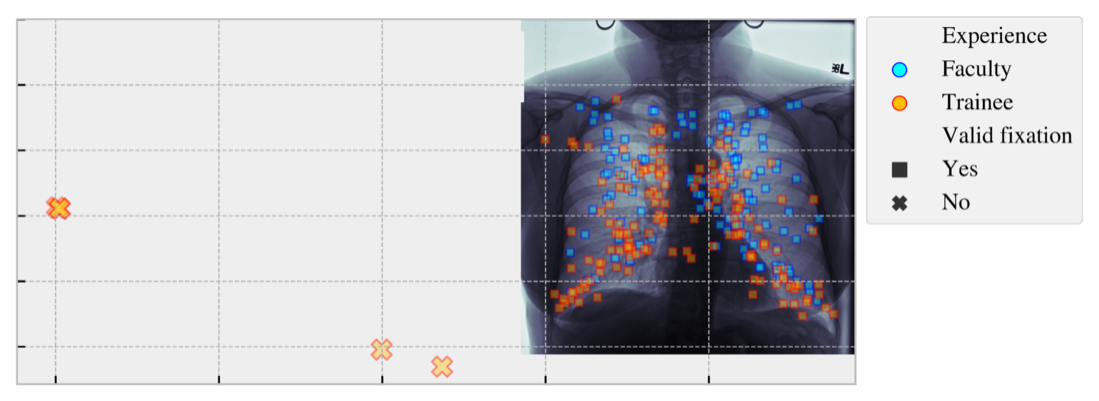
Example of eye-tracking fixations for 1 trial processed by the EyeLink software. The fixations illustrated include participants 1 (blue) and 2 (red) superimposed on the image displayed during the trial. The “invalid” fixations that were not successfully filtered out are shown as “x” markers and were manually removed during data processing.

In the first study, the EyeLink 1000 eye tracker and software were used to collect eye-tracking data [26]. A total of 2 participants—1 faculty member (9 years of faculty experience) and 1 resident (3 years of trainee experience)—observed a series of chest x-ray images, which contained a balanced class composition of normal scans (no abnormalities), abnormal scans (mass or nodule present), or abnormal scans with pleural effusion. A total of 110 trials (55 trials were studied by each participant) were recorded. We leveraged the EyeLink suite to remove most artifacts, such as blinks, from the eye-tracking data captured in each participant’s trial and manually filtered remaining artifacts, such as off-screen distractions left unprocessed (eg, the far-displaced fixations in Figure 2).

In the second study, a Tobii 5L eye tracker was used [27]. This second dataset included 8 participants (4 faculty with an average of 12.75 years of faculty experience and 4 trainees with 2.25 years of trainee experience), each scanning the same set of 27 images. The Tobii gaze data were unprocessed to evaluate the robustness of the proposed method to fixation postprocessing.

#### Common (Traditional) Features

To establish a necessary baseline to which the proposed methodology can compare, several attributes were established based on a meta-analysis done by van der Gijp et al [11] in 2017. The baseline is used for two main reasons: (1) traditional features are well-known and correlate with radiologist expertise, serving as a necessary reference point to evaluate our proposed method’s effectiveness, and (2) comparing our novel discretized vector encoding method against this baseline demonstrates the added value, improved classification accuracy, and robustness of our new approach. We separated those features based on if they required ground truthing of exams. Meanwhile, ground truthing of medical exams is costly and time-consuming as it involves manual annotation by experts, which also limits the scalability of the method. In addition, reliance on ground truth annotations can introduce biases and errors, as the annotations themselves might vary between experts, which limits applicability and transferability to real-world applications. Consequently, features that required knowledge of the image abnormalities’ ground truth location (ie, area of interest) were removed: fixation duration on the area of interest, number of fixations on the area of interest, and the time between trial start and the first fixation on the area of interest. This ensures that the proposed approach can be more easily and widely applied in clinical settings without the need for extensive preparatory work. It also helps mitigate potential variability and subjectivity in the training data, leading to more robust and generalizable models. Furthermore, by excluding features tied to known locations abnormally, we can better capture inherent differences in visual search patterns between experienced and less experienced radiologists.

Table 1 summarizes the remaining attribute names, descriptions, and expected association with levels of expertise. All features were used as originally defined except for coverage. Salient regions refer to areas of an image that are not part of a peripheral black background. This is typically necessary because users may be viewing scans with different amounts of background area. As noted previously, we used the Tobii gaze data without fixation postprocessing. For evaluating traditional features using fixations in the Tobii dataset, we substituted raw gaze data with fixation data. For purposes of clarity and brevity, we use fixations and gaze interchangeably for the remainder of the paper.

### Proposed Approach: Discretized Vector Encoding for Fixation Data

**Figure 3:**
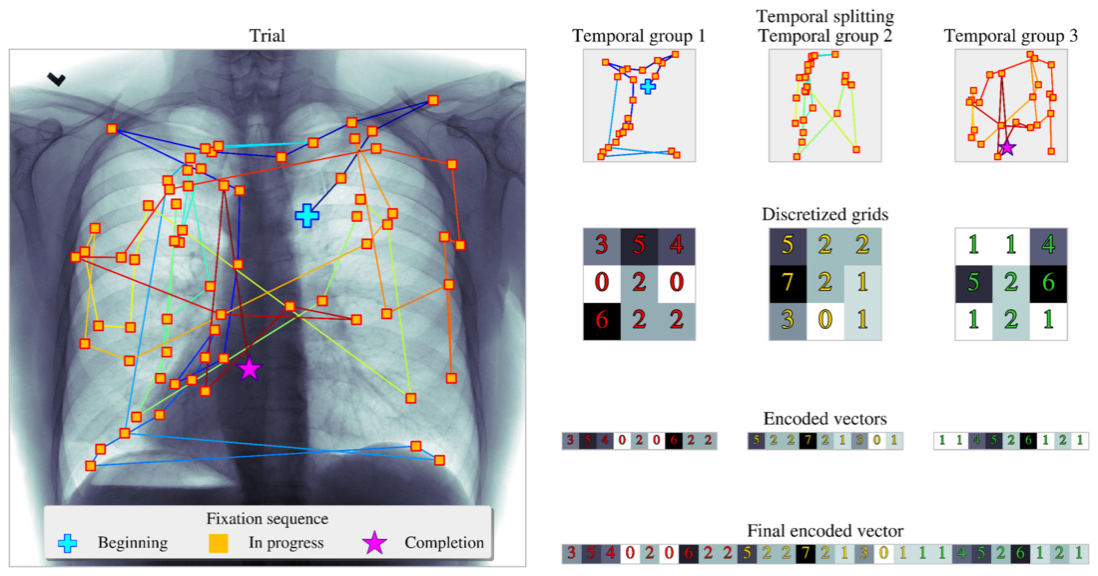
Proposed discretized vector encoding for fixation data. Bins 1, 2, and 3 capture fixations in a preserved spatial dimension across different temporal windows. Each row represents a temporal bin, and within each bin, the chest x-ray image is divided into spatial grids. The fixations are counted within each grid cell, providing a detailed representation of the radiologist’s visual search pattern over time.

Here, we describe the proposed method for directly using the fixation patterns as an alternative approach to using the current and previously described attributes in Table 1. The proposed strategy aims to extract information from fixations in the following 2 ways: first, geometric alignment: this involves mapping the coordinates of eye fixations on the chest x-ray images into a Cartesian grid. Each fixation is assigned to a specific grid cell based on its position, such as the Cartesian locations of the fixations when displayed on a chest x-ray image. Second, temporal order in which the fixations appear: the order in which fixations occur is crucial. Fixations are split into temporal bins, preserving the sequence of visual inspection. For each trial with recorded fixation data, we split the fixations into *t* number of temporal bins (each bin covers “total time divided by the number of bins in seconds”) or groups before counting the number of fixations captured within square grids or subdivisions of size *x*. Then, the *t* number of *x-*by*-x* grids is encoded into a single vector of size *1*-by*-(x×x×t).* The overall procedure is described in pseudocode in algorithm 1 (Textbox 1) and illustrated in Figure 3.

**Discrete vector encoding for fixation data.**

**Algorithm 1: Vector encoding for fixation data**

- Input
- n-Fixation coordinates of a single trial, F[n×2], number of x- and y-axis subdivisions, (x,y), number of temporal groups, (t)
- Output
- Encoded vector, V[1×(t×x×y)]
- Initialize
- Create array A[t×x×y] and centroids C[(x×y)×2] corresponding to the center of each grid subdivision (defined by second and third indices of A
- Evenly split fixations into t-groups, T = ([F1, F2, ⋯]1, ⋯, [⋯, F(n-1) Fn ]t)
- Procedure
- For *i*= 1 →>*t* do:
  *f* = *T*_i_
  For *j* = 1 → *len* (*f*) do:
  C^*^=*argmin* (|| *C*-*f*_j_ ||)
  *A* [i, *C*_x_^*^, *C*_y_^*^] += 1
  *V* = *vec*(*A*)
  Return *V*

In Figure 3, participant 1 inspects a single chest x-ray image; the processed fixations are captured as illustrated gold squares with red edges, with the first fixation labeled as a blue cross and the last fixation visualized as a magenta star. In this example, the fixations are split into 3 temporal segments (Step 1), in which 3-by-3 grids count the number of fixations within them (Step 2). Then, the proposed algorithm outputs the final encoding vector as the flattening and concatenation of the set of 3-by-3 grids (Step 3). For a given (square) grid of size *x* and *t* number of temporal segments, the final yielded output vector is of length, regardless of trial temporal duration. Segmenting the raw data into fixed temporal segments is one of the benefits of this approach and a strategy developed and imposed to generate consistent numbers of variables on the encoding output across different trials. As the number of fixations across each trial can vary between participants, fixing the number of temporal segments allows the capturing of trial duration while conforming to a prescribed number of grid subdivisions and temporal groups. For example, with Figure 3 as a reference, the final encoded vector will yield a vector with larger values therein for longer trials and yield a sparse vector (with lower values or values of zero) for shorter trials. Users can increase the fidelity of the grid and the number of time groups to represent a continuous spatiotemporal domain more closely. It is notable that the proposed methodology possesses the capability for tensor configuration for use in deep learning architecture by using *t* layers of grids. This tensor configuration is not studied in the paper due to the small sample size. The introduced technique is designed with more accessible or simpler classifiers in mind.

**Performance Metric and Simulation Setup**

To evaluate the discriminability of participants using the proposed approach, we use a stratified-fold cross-validation to calculate the AUC metric for several classification models, where each of the folds contains 5 trials from both levels of experience as the hold-out set. The study was performed on the data acquired by the EyeLink and Tobii equipment separately, and the following sections will contain an elaboration on their respective results. We performed cross-validations on a full-factorial configuration of 5, 7, 10, and 15 square grid subdivisions and 3, 5, 10, and 20 temporal groupings and selected the settings for each classifier that yielded the best results. In the presentation of these results, the average scores were calculated by computing the AUC metrics at the lowest level (data acquisition method, classifier, data type, feature extraction method, grid-size, temporal-group, and cross-validation seed) and averaged to the presented levels of granularity. Given the small sample size of 110 (EyeLink dataset) and 216 (Tobii dataset) trials, and high dimensionality in the chosen configurations (up to 4500 encoded variables in our study), there are available pathways that we have used to alleviate the effects of the curse of dimensionality present [28], such as principal component analysis (PCA) [29] and kernel principal component analysis (KPCA) [30]. The feature extraction and dimensionality reduction methods used include reducing the input data to 2 dimensions (with varying amounts of explained variance) and fixing the amount of variance explained to 50%, 90%, and 99% (with varying numbers of dimensions). These techniques were used not only to reduce the density of the data but also to introduce an additional preprocessing step that leverages the spectral decomposition of data collected from each participant.

Some of the major reasons for considering PCA and KPCA instead of the other alternatives include the following: PCA and KPCA are among the most popular method of dimensionality reduction; most technical practitioners, especially in the field of medicine, are familiar with PCA and KPCA; PCA and KPCA have rigorous mathematical properties and commonly used baseline methods in statistical analysis; and PCA and KPCA have relatively low computational complexity compared with many of the other shallow and deep alternatives.

All the codes were written using Python. The used libraries and versions are as follows: *matplotlib* (3.7.1), *seaborn* (0.12.2), *tqdm* (4.65.0), *scipy* (1.8.0), *scikit-learn* (1.0.2), *xgboost* (1.7.5), *GPy* (1.10.0), *numpy* (1.21.6), *pandas* (2.0.1), and *joblib* (1.2.0).

### Ethical Considerations

This study was conducted in full compliance with human participant research ethics and was reviewed and approved by the University of Texas Health San Antonio Institutional Review Board (20190533HU). All participants were fully informed about the purpose and procedures of the study, and informed consent was obtained before their inclusion. To ensure the privacy and confidentiality of participant data, all identifying information was removed to anonymize the dataset before analysis. Furthermore, participants were compensated $400 USD for their time and involvement in the study.

## Results

### Competing Algorithms and Training

In this study, we use the Gaussian process, logistic regression, and k-nearest neighbors classifiers from the *Scikit-learn* [31] package; the extreme gradient boosting (XGBoost) [32] tree-based ensemble classifier; and a modified AlexNet [33] deep learning classifier. The *Scikit-learn* classifiers were selected for their accessibility to users, while the XGBoost and AlexNet-like neural networks were chosen as more complex classifiers. The logistic regression, k-nearest neighbors, and XGBoost classifiers used *Scikit-learn*’s *StratifiedKFold* and *GridSearchCV* packages to train on the balanced accuracy loss function (also defined by *Scikit-learn*), while the Gaussian process methodology used Laplace approximation as detailed in their documentation [31]. Finally, the AlexNet-like classifier used sparse categorical cross-entropy [34] for training.

### EyeLink Dataset

**Figure 4:**
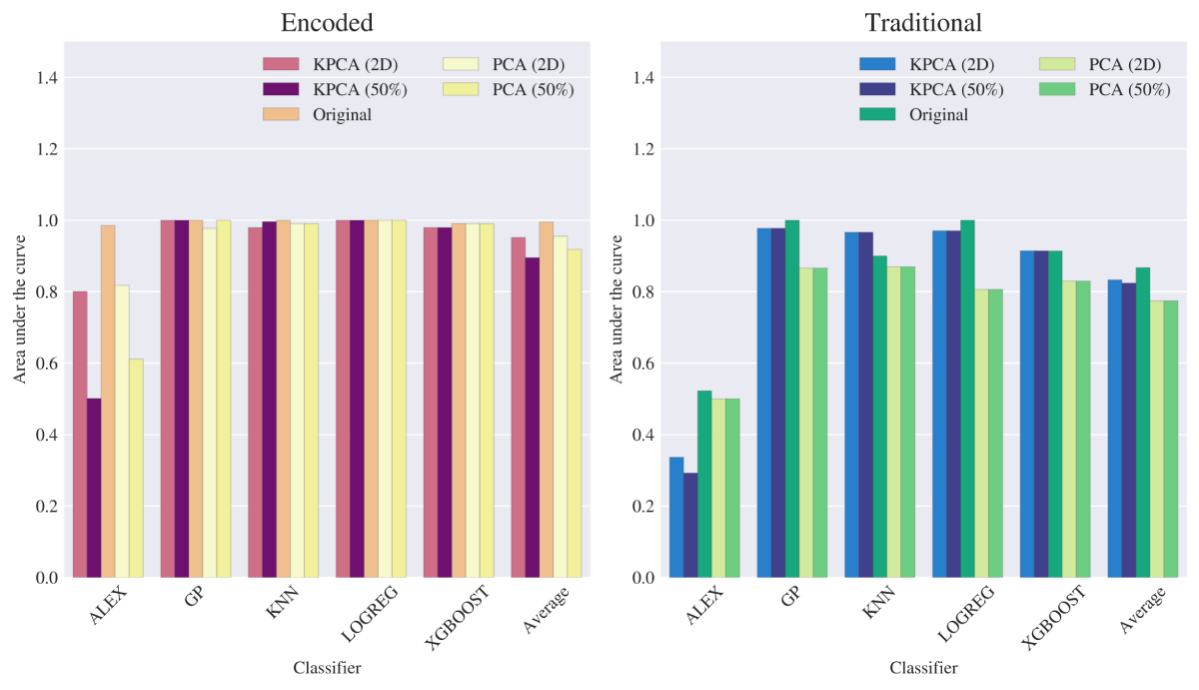
Numerical study results on the area under the curve metric reported for each classifier when consuming the EyeLink dataset, organized by the aggregated average of classifier, data type, and select feature extraction levels using the original dataset, principal component analysis (PCA), and kernel principal component analysis (KPCA). Alex: AlexNet-like neural network classifier; Average: average of all classifiers; GP: Gaussian process; KNN: k-nearest neighbors; LR: logistic regression; XGBoost: extreme gradient boosting;.

Figure 4 illustrates the average AUC across each classifier tasked with distinguishing between 2 participants (particularly between 2 levels of experience) using either the traditional or the proposed encoded data types (features). Along with the original data types, we include the average AUC of the classifiers based on the usage of select feature extraction configurations. The encoded features extracted from the raw dataset, shown on the left, illustrate a consistently high AUC score compared with the traditional features shown on the right, implying that the model performance for each classifier (except for certain feature extraction configurations of the AlexNet model) has high discriminatory power under optimal spatiotemporal encoding settings. 

We also present the AUC metrics from Figure 4 below in Table 2. The performance of the classifiers using the encoded data type consistently yielded higher discriminatory power than those using the traditional data type across all feature extraction methods. Encoding the fixation data into the proposed spatiotemporal elements provides more information each classifier can use to determine the experience level of a given participant more effectively than using traditional attributes. This table illustrates the original encoded data to possess the highest performance, with AUC scores consistently above 0.98 across all classifiers. However, usage of the traditional data yields roughly 0.522 at worst, as seen in the reported results for the AlexNet classifier. This trend of encoded data providing better results is also seen when using feature extraction; although a performance decrease is observable when reducing dimensions either through an information covariance matrix (PCA) or spatial relation (KPCA), the use of encoded data still outperforms those corresponding to the use of the traditional data. This suggests that the loss in information due to dimensionality reduction can be considered negligible in light of the benefits of using spatiotemporal encoding. The lower relative performance of the AlexNet-like classifier is likely caused by the number of training samples available in this study. The report on AUC in the table for the classifier is higher for the encoded data type, where it is observable that using the data without dimensionality reduction provides the best performance. This effect has been studied in Sumner and Alaeddini [35], in which neural networks already perform feature extraction throughout each present layer; this supportively evidences the reported results here, whereas (besides the small dataset) performing feature extraction beforehand may not provide enough information for the network to use its architecture to its fullest potential.

**Table 2:** Numerical tabulation of area under the curve scores across each classifier and data type and select feature extraction methods.

Feature extraction method and data type		Classifier for the EyeLink dataset, AUC^a^				
		AlexNet	Gaussian process	KNN^b^	LR^c^	XGBoost^d^
**KPCA^e^ (2D)**						
	Encoded	0.801	1.000	0.980	1.000	0.980
	Traditional	0.336	0.978	0.967	0.970	0.914
**KPCA (50%)**						
	Encoded	0.501	1.000	0.996	1.000	0.980
	Traditional	0.292	0.978	0.967	0.970	0.914
**Original**						
	Encoded	0.985	1.000	1.000	1.000	0.991
	Traditional	0.522	1.000	0.900	1.000	0.914
**PCA^f^ (2D)**						
	Encoded	0.818	0.978	0.991	1.000	0.991
	Traditional	0.500	0.866	0.870	0.806	0.830
**PCA (50%)**						
	Encoded	0.611	1.000	0.991	1.000	0.991
	Traditional	0.500	0.866	0.870	0.806	0.830

^a^AUC: area under the curve.

^b^KNN: k-nearest neighbor.

^c^LR: logistic regression.

^d^XGBoost: extreme gradient boosting.

^e^KPCA: kernel principal component analysis.

^f^PCA: principal component analysis.

**Figure 5:**
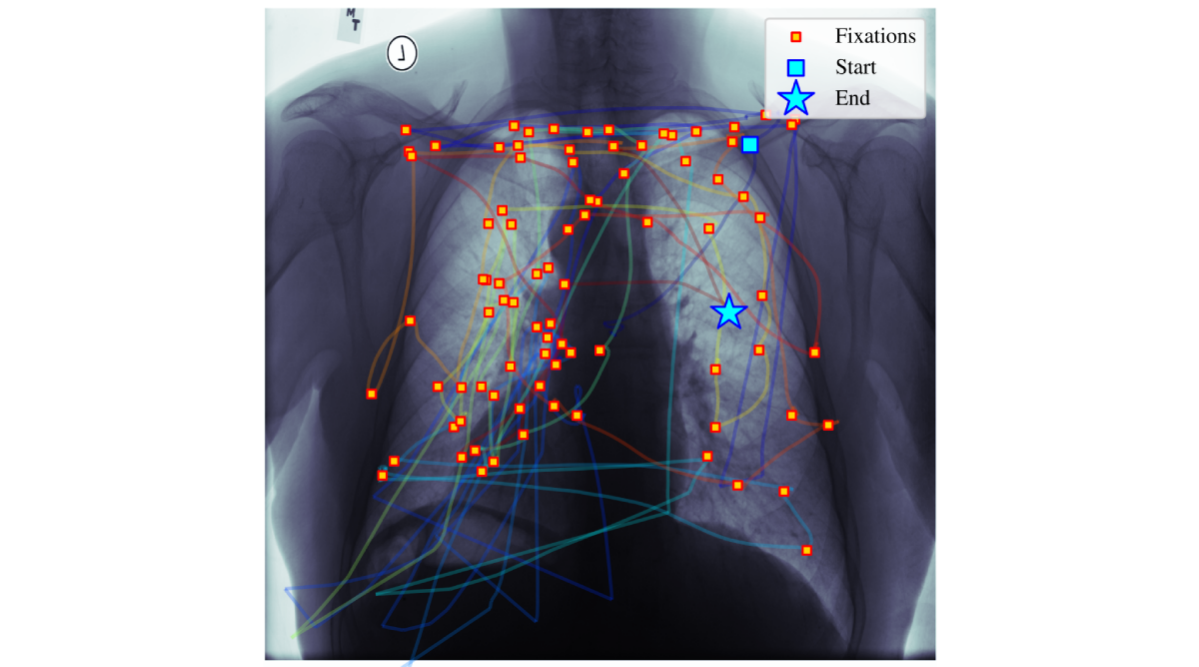
Scan of chest x-ray by participant 1 (faculty).

**Figure 6:**
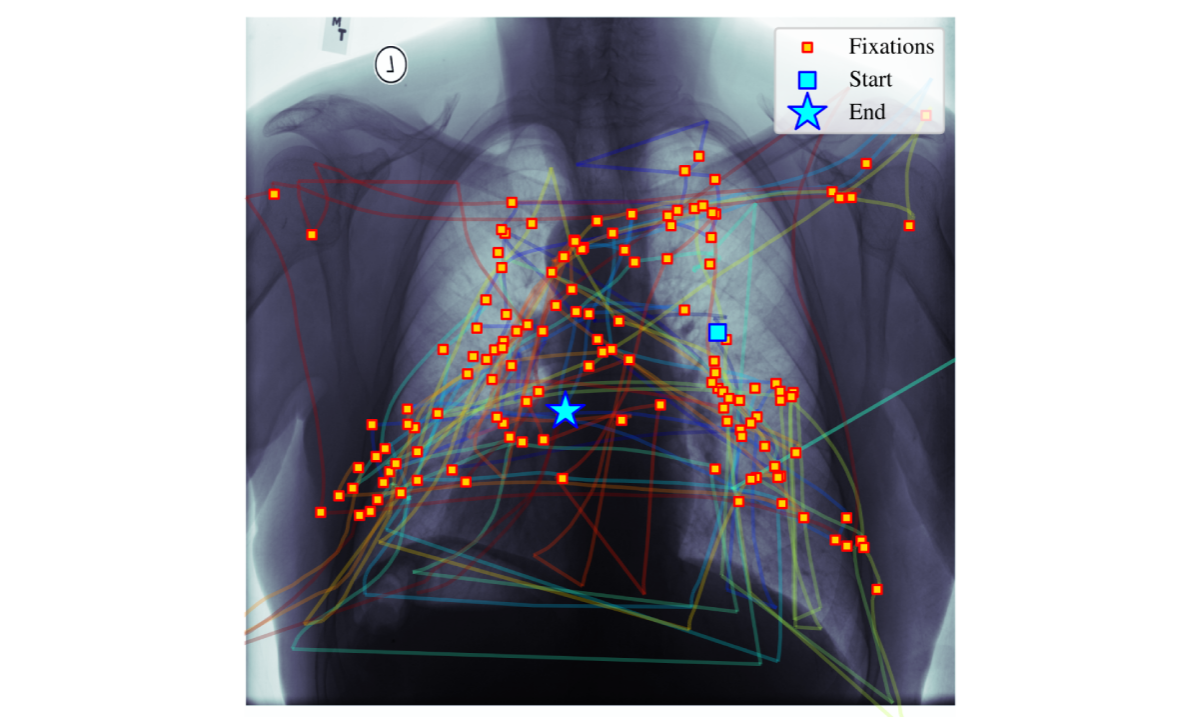
Scan of chest x-ray by participant 2 (trainee).

By using the encoded vectors for classification, differences in eye-tracking patterns can more consistently be distinguished between the 2 participants. Figures 5 and 6 illustrate one such difference in search pattern behavior. The more experienced participant (participant 1, Figure 5) shows a more uniformly distributed search pattern across the chest x-ray. In contrast, the less-experienced participant (participant 2, Figure 6) focuses on regions where they suspect abnormalities. It is clear from a visual inspection that the behavior between these participants is markedly different and using the correct spatiotemporal configurations to capture the differences between the 2 participant’s behavior by leveraging the proposed methodology (as reported numerically in Table 2) provides a consistent improvement of classification accuracy.

### Tobii Dataset

**Figure 7:**
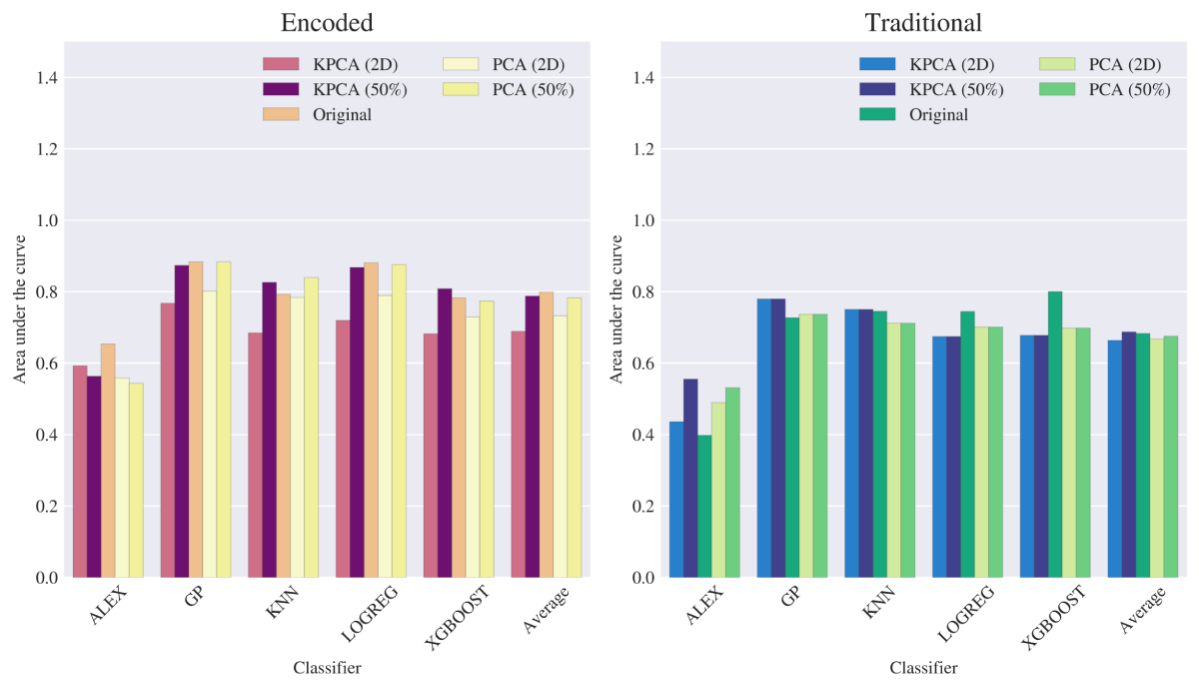
Numerical study results on the area under the curve metric reported for each classifier when consuming the Tobii dataset, organized by the aggregated average of classifier, data type, and selected feature extraction levels using the original dataset, principal component analysis (PCA), and kernel principal component analysis (KPCA). Alex: AlexNet-like neural network classifier; Average: average of all classifiers; GP: Gaussian process; KNN: k-nearest neighbors; LR: logistic regression; XGBoost: extreme gradient boosting;.

We have performed the same analysis on the data acquired using Tobii eye-tracking equipment. It is notable that although the AUC scores from the EyeLink dataset are consistently high, natural anticipations allow one to observe more variation in classifier performance when more individual participants (classified as either a more-experienced faculty or less-experienced trainee) are introduced to the study. Figure 7 illustrates a report on AUC in a similar fashion to that in Figure 4, with lower scores across all classifying models for both data types. As seen in Figure 4, Figure 7 also suggests that the best performance for the encoded data on average is attained when using it without feature extraction, although, for several cases, we can observe that some form of feature extraction yields better results than their respective traditional dataset counterparts.

When inspecting Tables 3, we can numerically inspect the average (and variance of) AUC, *F*_1_-score, accuracy, specificity, and sensitivity of each classifier when consuming each data type in both datasets. Within the Tobii dataset, the encoded data type generally outperformed (shown in italics) the traditional data type across most metrics and models. Although the encoded data type that was consumed within the Tobii dataset possessed more discriminatory capability than that in the traditional data, the performance gap was less pronounced than those observable in the EyeLink dataset. For example, the Tobii average (and variance) AUC scores for the encoded data type ranged from 0.55 (0.05) to 0.82 (0.04), while the traditional data type ranged from 0.51 (0.07) to 0.76 (0.05), and the EyeLink average (variance) AUC scores for the same data types ranged from 0.63 (0.07) to 1.0 (0.0) and from 0.52 (0.08) to 0.96 (0.01), respectively. There is a consistent trend across datasets that support the encoded data are capable of providing higher values of accuracy and performance; the *F*_1_-score for the Gaussian process, k-nearest neighbors, logistic regression, and XGBoost were consistently higher when using the encoded data than when using traditional attributes in classification. This highlights the ability of the proposed encoding procedure to improve the balance between precision and recall in the classifiers and, as a result, the overall effectiveness of each model’s predictions. In terms of specificity, the encoded data type is also shown to have a competitive edge in boosting a classifier’s ability to correctly identify true negative class labels (experienced participants). As seen in Table 3, the average range of improvement lies between 0.01 to 0.04 for the EyeLink dataset and between –0.2 to +0.15 for the Tobii dataset; the negative value of the improvement is seen with the AlexNet-like model, which, as explained before, may have difficulty fitting well for classification on small datasets, made more difficult by the variation in subconscious behavior between participants that are recorded in spatiotemporal encodings by the proposed methodology.

**Table 3:** Numerical tabulation of mean and variance area under the curve, *F*_1_-score, accuracy, specificity, and sensitivity across each data acquisition method, classifier, and data type.

Metric and classifier		Data acquisition system				
		EyeLink			Tobii	
		Data type			Data type	
		Encoded, average (SD)	Traditional, average (SD)	Encoded, average (SD)		Traditional, average (SD)
**Area under the curve**						
	AlexNet	*0.63 (0.07)* ^a^	0.52 (0.08)	*0.55 (0.05)*		0.51 (0.07)
	GP^b^	*1 (0)*	0.96 (0.01)	*0.82 (0.04)*		0.76 (0.05)
	KNN^c^	*0.96 (0.01)*	0.93 (0.01)	0.73 (0.05)		*0.74 (0.04)*
	LR^d^	*1 (0)*	0.93 (0.01)	*0.82 (0.04)*		0.71 (0.08)
	XGBoost^e^	*0.97 (0.0)*	0.91 (0.01)	*0.73 (0.05)*		0.71 (0.05)
***F*_1_-score**						
	AlexNet	*0.43 (0.1)*	0.41 (0.09)	*0.39 (0.1)*		0.23 (0.1)
	GP	*0.98 (0)*	0.90 (0.03)	*0.73 (0.07)*		0.71 (0.05)
	KNN	*0.90 (0.03)*	0.88 (0.02)	0.61 (0.1)		*0.71 (0.04)*
	LR	*0.98 (0)*	0.82 (0.04)	*0.74 (0.06)*		0.58 (0.09)
	XGBoost	*0.96 (0)*	0.87 (0.02)	*0.68 (0.07)*		0.67 (0.05)
**Accuracy**						
	AlexNet	*0.52 (0.02)*	0.49 (0.04)	*0.51 (0.0)*		0.5 (0.01)
	GP	*0.99 (0.0)*	0.93 (0.01)	*0.76 (0.03)*		0.7 (0.03)
	KNN	*0.93 (0.01)*	0.9 (0.01)	0.69 (0.03)		*0.7 (0.02)*
	LR	*0.98 (0.0)*	0.88 (0.01)	*0.77 (0.03)*		0.64 (0.03)
	XGBoost	*0.96 (0.0)*	0.9 (0.01)	*0.72 (0.03)*		0.67 (0.03)
**Specificity**						
	AlexNet	*0.43 (0.22)*	0.42 (0.22)	0.46 (0.16)		*0.66 (0.12)*
	GP	*0.98 (0)*	0.97 (0.01)	*0.80 (0.04)*		0.65 (0.07)
	KNN	*0.97 (0.01)*	0.93 (0.01)	*0.76 (0.06)*		0.67 (0.04)
	LR	*0.99 (0.0)*	0.97 (0.01)	*0.81 (0.04)*		0.67 (0.08)
	XGBoost	*0.95 (0.01)*	0.92 (0.01)	*0.76 (0.05)*		0.63 (0.08)
**Sensitivity**						
	AlexNet	*0.64 (0.22)*	0.57 (0.2)	*0.56 (0.17)*		0.32 (0.12)
	GP	*0.99 (0)*	0.89 (0.04)	0.72 (0.07)		*0.76 (0.05)*
	KNN	*0.89 (0.05)*	0.87 (0.03)	0.61 (0.1)		*0.74 (0.04)*
	LR	*0.98 (0.01)*	0.77 (0.06)	*0.72 (0.06)*		0.6 (0.09)
	XGBoost	*0.98 (0)*	0.86 (0.03)	0.68 (0.07)		*0.71 (0.05)*

^a^Superior values are italicized.

^b^GP: Gaussian process.

^c^KNN: k-nearest neighbor.

^d^LR: logistic regression.

^e^XGBoost: extreme gradient boosting.

## Discussion

### Principal Findings

In this study, we demonstrated the capacity of eye-tracking technology, combined with machine learning algorithms, to discriminate between radiologists’ experience levels. For this purpose, we developed a novel feature encoding technique where fixations are first spatially arranged according to their Cartesian coordinates on chest x-ray images and temporally ordered. The fixations are then subdivided into predefined temporal bins, and within each bin, we count the number of eye fixations within each subdivision. These counted bins are then concatenated to form a vector encoding to be used as feature input for machine learning algorithms. Our experiments showed that the discretized vector encoding creates discriminative features that are not captured by conventional metrics. Using the encoding approach allows classifiers to better distinguish between participants in terms of experience level, which highlights performance gains (when compared with using traditional features for discrimination) of 6.9%, 7.11%, 9.14%, 9.59%, and 5.61% for AUC, accuracy, *F*_1_-score, sensitivity, and specificity, respectively, aggregated across both EyeLink and Tobii datasets in Table 3. The Tobii dataset exhibits a lower performance gain (6.41%, 7.48%, 8.62%, 5.11%, and 9.45%) than observed using the EyeLink dataset (7.29%, 6.83%, 9.54%, and 13.13%) due to using a more diverse roster of participants; however, the trend in using the proposed eye-tracking encoding approach possessing the competitive edge is still present, highlighting the effectiveness of spatiotemporal assortment in the introduced method. These results validate our initial hypothesis that when appropriately encoded, eye-tracking data can provide nuanced insights into the difference between radiologist’s expertise levels.

We can also observe the perceptual strategies radiologists use during diagnostic evaluations. Previous research has often focused on more general eye-tracking metrics without leveraging the full potential of machine learning to analyze the data. For example, studies by van der Gijp et al [11] and Waite et al [3] explored how visual search patterns correlate with diagnostic accuracy and expertise. With the help of the proposed encoding method, such machine learning models can be developed to determine expertise level and has the potential to identify and track potential features from eye fixations or gaze fixations.

### Limitations

While our study has shown promising results and potential benefits, it is important to acknowledge limitations that may have a degree of effect on our findings. One such limitation is sample size; across both the EyeLink and Tobii datasets, there were 2 participants in one study (EyeLink) and 8 participants in the other (Tobii), with both containing small numbers of images scanned by each participant. Another condition involves the variation in data acquisition. In total, 2 different eye-tracking devices (EyeLink and Tobii) were used for data collection, and while serving the same overarching purpose of collecting data, some additional variability in the findings are notably attributed to the usage of 2 different hardware-software configurations. Another important consideration includes the difference in traditional feature sets between the EyeLink and Tobii datasets. Coupled with data acquisition differences, some features from the EyeLink software were not congruent with the Tobii dataset, such as the usage of fixations (EyeLink) versus gaze (Tobii). When applying the encoding approach to these datasets, the Tobii dataset had larger yielded values in each output vector. This did not affect the results substantially; however, it underscores the challenge of directly comparing data from 2 sources. One final consideration was our decision to remove certain metrics related to the location of abnormalities in chest x-rays as features in the traditional data type during performance evaluation. For example, we did not consider the time to first fixation on the region of abnormality. This and other like attributes possess statistical significance in previous works; however, their inclusion necessitates extensive labeling, validation, and other processing in order to establish ground truth information for each image scanned by each participant.

### Conclusions

Despite the limitations above, this study holds significant promise and offers a range of benefits worthy of attention and consideration for use. By extracting spatiotemporal features from eye-tracking data, the proposed approach has demonstrated the capacity to differentiate users based on eye-tracking behavior alone instead of traditional methods and can be extended for use in fields ranging from medical to educational. The approach enables discriminability between users and offers a pathway to gaining deeper insights into generalized differences between levels of expertise. By exploring these pathways, this approach holds the potential to establish more effective educational programs that can assist users optimize their search patterns. Respective to the study conducted, by providing support to radiologists to find abnormalities quickly and accurately in chest x-rays, our approach seeks to reduce perceptual errors in medical diagnoses. In fields where the development of unique and precise search patterns is important, the proposed approach offers a valuable source of knowledge transfer. Transmission of expertise from more-experienced individuals to less-experienced individuals can be facilitated and result in increased streamlining during the learning process and yield more efficient and accurate search patterns. The potential benefits can apply to professionals and trainees or students alike.

In summary, we have shown the potential for spatiotemporal features extracted from eye-tracking data to be useful in discriminating between radiologists of different skill levels and opening the door to improving education. We plan to augment this research by increasing the number of radiologists to demonstrate generalizability and exploring additional types of spatiotemporal analyses. The implications of our findings extend beyond radiology, suggesting that similar methodologies could revolutionize training and assessment protocols in various fields that rely on visual cognition like aviation and ground transportation. Further research could explore the integration of these techniques into real-time training tools, potentially transforming educational paradigms in professions requiring visual expertise.

## Abbreviations

AUC: area under the curve
IRB: institutional review board
PCA: principal component analysis
KPCA: kernel principal component analysis
XGBoost: extreme gradient boosting

## Data Availability

Data collected and analyzed in this study are available upon request. Interested researchers can obtain the data by completing a data request form, which includes their planned project outline and the details of the person responsible for handling the data. This process ensures that the data are used responsibly and for scientifically valid purposes. The request forms are reviewed on a case-by-case basis to ensure alignment with ethical and research standards. Images and annotations were obtained from the National Institutes of Health Clinical Center and can be downloaded on the web [36]. The Institutional Review Board restricts sharing of the gaze data. In case further information, please contact KC (clarkkl@uthscsa.edu).
